# Microstructural Characterization and In Vitro–In Vivo Evaluation of Drug Release and Permeation in Goupi Plaster

**DOI:** 10.3390/pharmaceutics18050524

**Published:** 2026-04-25

**Authors:** Jia Liu, Tong Guan, Ailin Zhang, Yutong Liu, Zhixin Yang, Feng Guan, Weinan Li, Yanhong Wang

**Affiliations:** 1 School of Pharmacy, Heilongjiang University of Chinese Medicine, Harbin 150040, China; liujiaa0810@163.com (J.L.);; 2 Key Laboratory of Basic and Application Research of Beiyao, Heilongjiang University of Chinese Medicine, Ministry of Education, Harbin 150040, China; 3 College of Pharmacy, Harbin University of Commerce, Harbin 150028, China

**Keywords:** Goupi plaster (GP), transdermal delivery, in vitro release, skin permeation, microdialysis, pharmacokinetics

## Abstract

**Background/Objectives**: Goupi plaster (GP) is a traditional black plaster composed of a biphasic fibrous–oil matrix containing multiple bioactive compounds, and it has been widely used for the treatment of musculoskeletal disorders. Representative active compounds include sinomenine, osthole, cinnamaldehyde, and imperatorin, which exhibit anti-inflammatory and analgesic effects. However, due to its heterogeneous matrix structure and multi-component nature, the pharmaceutical delivery behavior of GP remains difficult to evaluate using conventional methods. Therefore, this study aimed to establish an integrated structure–release–permeation–pharmacokinetic evaluation framework to systematically characterize the transdermal delivery behavior of GP. **Methods:** GP was evaluated using multi-level analysis, including microstructural imaging (FESEM), in vitro release, ex vivo skin permeation, and in vivo dual-site microdialysis. Four representative bioactive compounds (sinomenine, osthole, cinnamaldehyde, and imperatorin) were selected as marker compounds. Release data were fitted to kinetic models, and structure–release relationships were examined using the Higuchi release constant (*k_h_*). Skin-barrier alterations were assessed by attenuated total reflectance–Fourier transform infrared spectroscopy (ATR–FTIR) and differential scanning calorimetry (DSC). Local concentrations in subcutaneous (SC) and intra-articular (IA) compartments were measured by ultra-performance liquid chromatography–tandem mass spectrometry (UPLC–MS/MS) to explore potential in vitro–in vivo correlation (IVIVC). **Results**: FESEM revealed a fibrous–oil network structure. GP exhibited sustained, diffusion-dominated release, with *k_h_* = 0.9908–0.9977 and Korsmeyer–Peppas (K–P) release exponents (*n*) = 0.61–0.66, differing from active pharmaceutical ingredient (API) controls. Fiber area fraction and fiber length density showed negative correlations with *k_h_* (*r* = −0.91 to −0.99); ex vivo permeation profiles varied among compounds, and ATR–FTIR and DSC analyses showed moderate changes in skin-barrier properties. Dual-site microdialysis demonstrated sustained local exposure, and a positive relationship was observed between in vitro release and in vivo concentrations. **Conclusions**: This study establishes an integrated structure–release–permeation–pharmacokinetic evaluation framework for traditional black plaster systems. The observed IVIVC is descriptive rather than predictive, reflecting a trend-level association under the current experimental conditions. These findings highlight the importance of integrating in vitro release, skin permeation, and local pharmacokinetics for understanding drug delivery behavior in complex transdermal matrix systems, and provide a methodological basis for quality consistency evaluation of traditional black plaster formulations.

## 1. Introduction

Black plaster is a traditional external dosage form prepared by refining vegetable oil in which herbal materials have been extracted together with lead oxide at high temperature, and then spreading the resulting matrix onto a backing material for topical application. With a long history of clinical use, it represents one of the classical external preparations in traditional Chinese medicine [1]. Goupi Plaster (GP) is a representative black plaster formulation that is widely used for the treatment of musculoskeletal disorders, such as arthralgia, soft tissue injury, and knee osteoarthritis [2]. Previous studies have shown that the black plaster matrix forms a double continuous phase system composed of a viscous oil phase and a cross-linked network of branched soap crystal fibers, which together constitute a biphasic fibrous–oil matrix structure [3]. This structure provides the plaster with strong adhesion, high drug-loading capacity, and sustained-release properties. GP contains a complex mixture of bioactive compounds derived from herbal materials. Among these, sinomenine, osthole, cinnamaldehyde, and imperatorin are commonly recognized as representative marker compounds with reported anti-inflammatory, analgesic, and cartilage-protective activities [4,5,6,7,8,9]. Importantly, these components differ substantially in their physicochemical properties, suggesting that their transdermal behavior may be influenced by multiple factors, including individual molecular characteristics and the structural features of the formulation, potentially contributing to multi-component synergistic therapeutic outcomes. Therefore, GP can be regarded as a complex transdermal delivery system composed of a fibrous–oil matrix and multiple pharmacologically active compounds.

Compared with modern transdermal delivery systems such as synthetic patches, nanoemulsions, and hydrogels, GP exhibits distinctive characteristics. Modern transdermal systems are typically designed as homogeneous matrices containing a single active pharmaceutical ingredient, with well-defined release kinetics and controllable permeation behavior. In contrast, GP is a heterogeneous system in which multiple active compounds are embedded within a fibrous–oil biphasic matrix, forming a structurally complex drug reservoir [3]. This structural complexity may contribute to multi-component synergistic therapeutic effects and sustained drug release behavior, and the formulation is relatively cost-effective and simple to manufacture [10,11]. However, these characteristics also present challenges, including difficulties in standardization, batch-to-batch variability, and the lack of systematic pharmaceutical evaluation methods [12]. Therefore, although GP has been widely used in clinical practice, its drug delivery behavior has largely been evaluated empirically rather than through quantitative pharmaceutical analysis. From a drug delivery perspective, establishing a scientific evaluation framework for such a complex matrix system is essential for understanding its delivery behavior and ensuring quality consistency [13,14,15,16].

For transdermal delivery systems, drug absorption at the target site depends not only on drug release from the matrix but also on permeation across the skin barrier. Therefore, local drug exposure is associated with both drug release from the matrix and permeation across the skin barrier [17,18,19,20]. In complex matrix systems such as black plaster, this process becomes more complicated due to the heterogeneous fibrous–oil biphasic matrix and the presence of multiple active compounds with different physicochemical properties, making it difficult to directly correlate in vitro release behavior with in vivo drug exposure [21]. From a pharmaceutical and regulatory perspective, establishing an in vitro–in vivo correlation (IVIVC) is important for formulation optimization, quality control, and batch-to-batch consistency evaluation [22,23]. However, for traditional black plaster systems, there is currently a lack of systematic studies that quantitatively link matrix structure, drug release, transdermal permeation, and in vivo pharmacokinetics. Therefore, developing an integrated evaluation approach that connects these processes is of great importance for the pharmaceutical characterization of black plaster systems.

Therefore, the objective of this study was to establish an integrated structure–release–permeation–pharmacokinetic evaluation framework for the systematic characterization of GP as a traditional black plaster system. This study further sought to explore whether local tissue exposure of active compounds from GP is associated with in vitro release behavior and skin-barrier permeation, as well as potential relationships between in vitro release profiles and in vivo local pharmacokinetics. The novelty of this study lies in the methodological integration of microstructural characterization, release kinetics, transdermal permeation, and in vivo microdialysis pharmacokinetics into a unified evaluation framework for black plaster systems. This integrated framework provides a quantitative approach for pharmaceutical evaluation, quality consistency assessment, and formulation optimization of traditional black plaster formulations. Importantly, this study focuses on exploring structure–function correlations among microstructure, release, permeation, and pharmacokinetic behavior in GP, rather than establishing a definitive mechanistic model, with emphasis on framework development and correlation analysis for complex fibrous–oil biphasic matrix transdermal systems.

## 2. Materials and Methods

### 2.1. Chemicals

The reagents for this experiment included six batches of GP (Batch 1–Batch 6), obtained from a licensed manufacturer (Guiyang Jirentang Pharmaceutical Co., Ltd., Guiyang, Guizhou, China). Reference standards of sinomenine, osthole, cinnamaldehyde and imperatorin were purchased from the National Institutes for Food and Drug Control (Beijing, China). Distilled water was obtained from Watsons Food & Beverage Co., Ltd. (Guangzhou, China). LC–MS grade methanol and acetonitrile were purchased from Merck (Darmstadt, Germany). LC–MS grade formic acid was purchased from West Asia Reagent Co., Ltd. (Chengdu, China). Analytical grade petroleum ether and ethyl acetate were obtained from Fuyu Fine Chemical Co., Ltd. (Tianjin, China).

### 2.2. Experimental Animals and Treatment Protocols

Full-thickness abdominal skin used for the ex vivo permeation study, attenuated total reflectance–Fourier transform infrared spectroscopy (ATR–FTIR) and differential scanning calorimetry (DSC) were obtained from male Sprague-Dawley rats (220–250 g), provided by the Laboratory Animal Center of Heilongjiang University of Traditional Chinese Medicine (Harbin, China). The rats were housed under standard laboratory conditions and had free access to standard chow and water. Standard rat chow was purchased from Liaoning Changsheng Biotechnology Co., Ltd. (Benxi, Liaoning, China), containing approximately ≥18% crude protein, ≥4% crude fat, ≤5% crude fiber, with moisture ≤10%, calcium 1.0–1.8%, and phosphorus 0.6–1.2%. The diet also contained essential vitamins (vitamins A, D, E, K, and B group) and trace elements (Fe, Mn, Cu, and Zn).

A total of twelve healthy male New Zealand white rabbits (20–24 weeks, 2.5 ± 0.2 kg) were obtained from the Laboratory Animal Center of Heilongjiang University of Traditional Chinese Medicine (Harbin, China). Rabbits were housed in a temperature-controlled environment (25 ± 2 ℃, relative humidity 55 ± 5%) with a 12/12 h light/dark cycle, and had free access to standard laboratory chow and water. All animals were acclimated for one week prior to experimentation. Standard rabbit chow was purchased from Liaoning Changsheng Biotechnology Co., Ltd. (Benxi, Liaoning, China). The diet contained approximately ≥14% crude protein, ≥3% crude fat, and 10–15% crude fiber, with moisture ≤11%, calcium 1.0–1.5%, and phosphorus 0.5–0.8%. The diet also contained essential vitamins (vitamins A, D, E, K, and B group) and trace elements (Fe, Mn, Cu, and Zn).

The experimental protocol was approved by the Animal Care and Ethics Committee of Heilongjiang University of Traditional Chinese Medicine (approval number: 2018102301), and all procedures were conducted in accordance with the NIH Guidelines for the Care and Use of Laboratory Animals (USA). The animals were randomly assigned to six groups using a random number table, corresponding to six GP batches (Batch 1–Batch 6). Within each rabbit, microdialysis probes were simultaneously implanted into the subcutaneous (SC) tissue and the intra-articular (IA) cavity, thereby enabling paired dual-site measurements within the same animal. Dialysate samples from both sites were collected over multiple time points and analyzed for sinomenine, osthole, cinnamaldehyde, and imperatorin.

Blinding was not applied during the experiment because drug concentrations were quantified using an automated ultra-performance liquid chromatography–tandem mass spectrometry (UPLC–MS/MS) method.

### 2.3. Microstructural Characterization of GP by FESEM

#### 2.3.1. Pretreatment of GP

Six batches of GP (Batch 1–Batch 6) were analyzed, and three samples were prepared for each batch (*n* = 3 per batch). To investigate the internal structure of GP, the thickened vegetable oil matrix was first removed. Refrigerated GP samples were cut into 5 mm × 5 mm squares after removing the backing layer. Each sample was placed in a 250 mL conical flask and fully immersed in 100 mL of ethyl acetate. The flask was covered and left to soak at room temperature for 20 h. The ethyl acetate was then replaced with fresh solvent, and the flask was transferred to a 35 °C constant temperature water bath shaker at 50 rpm for 12 h, followed by a 12 h standing period. This solvent replacement was repeated every 12 h until the solution appeared colorless, over a total duration of 2 days. This solvent extraction procedure was performed solely for microstructural visualization and was not applied to samples used in release, permeation, or in vivo experiments. Therefore, the quantitative structural descriptors derived from field-emission scanning electron microscopy (FESEM) images should be interpreted as correlational parameters rather than direct representations of drug diffusion pathways.

#### 2.3.2. Microstructural Observation of Pretreated GP

The pretreated GP samples were sputter-coated with a thin layer of gold and observed using FESEM to characterize their microstructure [3,24,25].

#### 2.3.3. Quantitative Microstructural Analysis

FESEM images of all six GP batches (*n* = 6) were processed using Fiji (ImageJ, version 1.53k), an open-source image analysis software developed by the National Institutes of Health (Bethesda, MD, USA). Images were processed for background removal and local contrast enhancement, and then binarized to obtain pore and fiber layers. The fiber phase was skeletonized for length measurement. For each batch, three representative FESEM images were selected for analysis (*n* = 3 images per batch). To avoid selection bias, each image was divided into four fixed, equal-area regions of interest (ROIs), and the parameters were calculated for each ROI and averaged to obtain image-level values.

### 2.4. Analysis of GP by UPLC–MS/MS

#### 2.4.1. Chromatographic Conditions

Chromatographic separation was performed on a Waters ACQUITY UPLC^®^ BEH C18 column (2.1 × 100 mm, 1.7 μm, Milford, MA, USA). The mobile phase consisted of acetonitrile (B) and water containing 0.1% formic acid (C), with a flow rate of 0.3 mL·min^−1^. The column temperature was maintained at 30 °C, the sample room temperature at 15 °C, and the injection volume was 2 μL. The gradient elution program was as follows: 10–95% B at 0–8 min, 95–10% B at 8–13 min, and 10% B at 13–15 min [4].

#### 2.4.2. Mass Spectrometry Conditions

Quantitative analyses were performed on a Waters ACQUITY UPLC–TQD triple quadrupole mass spectrometer equipped with an electrospray ionization (ESI) source operated in positive ion mode. Instrumental parameters were set as follows: capillary voltage, 2.7–3.0 kV; desolvation temperature, 350–500 °C; and cone voltage optimized for each analyte. Detailed multiple reaction monitoring (MRM) transitions and parameters are summarized in Table 1 [4].

#### 2.4.3. Method Validation for UPLC–MS/MS Analysis

The UPLC–MS/MS method was validated in terms of linearity, precision, accuracy (expressed as recovery), repeatability, and stability. Calibration curves were constructed over appropriate concentration ranges for different sample types. The lowest calibration point meeting acceptable precision and accuracy criteria was defined as the lower limit of quantification for each analyte.

For in vitro-related samples, including content determination, in vitro release samples, and ex vivo permeation receptor samples, all analytes showed good linearity within their respective calibration ranges, with correlation coefficients greater than 0.992. The regression equations are summarized in Table S1. The relative standard deviation (RSD) values for precision, repeatability, and stability were all below 1.5%, and recoveries were within acceptable ranges with low variability, indicating good accuracy and stability of the method. Detailed validation results are presented in Table S2.

For in vivo microdialysis samples, calibration curves were established at lower concentration levels suitable for microdialysis analysis, and dialysate samples were analyzed by direct injection. The method also showed good linearity in biological samples, with correlation coefficients greater than 0.9989. The regression equations are listed in Table S3. The RSD values for precision and stability were all below 2.0%, and recoveries showed low variability. Detailed validation results are presented in Table S4, demonstrating that the method was suitable for quantitative analysis of microdialysis samples.

Method suitability evaluation demonstrated that the UPLC–MS/MS method exhibited acceptable linearity, precision, accuracy, and stability across different sample matrices. Therefore, the method was considered suitable for the quantitative determination of GP content, in vitro release samples, ex vivo permeation samples, and in vivo microdialysis samples.

### 2.5. Quantitative Analysis of Sinomenine, Osthole, Cinnamaldehyde and Imperatorin of GP

Mixed stock solutions were prepared by dissolving sinomenine (52.00 μg·mL^−1^) and imperatorin (125.00 μg·mL^−1^) in methanol, and separately dissolving osthole (100.50 μg·mL^−1^) and cinnamaldehyde (78.00 μg·mL^−1^) in methanol. Working solutions for calibration were obtained by serial dilution of the stock solutions with methanol.

To prepare the test solution, 2.00 g of GP were accurately weighed and evenly spread in separate beakers. Then, 40 mL of methanol solution was added to each beaker to ensure full contact with the plaster and the solvent. The mixture was ultrasonicated for 3 h and allowed to stand. It was then placed in a refrigerator at 4◦C for 48 h, filtered while cold. This filtration process was repeated three times until a clear, slightly oily filtrate was obtained. The filtrates were combined and evaporated under reduced pressure. Finally, the sample was filtered with a 0.45 μm filter membrane to obtain the test solution [4]. Quantification of sinomenine, osthole, cinnamaldehyde, and imperatorin was performed under the chromatographic conditions specified in Section 2.4. In addition, the effective surface area of the plaster was experimentally determined, showing that 2.00 g of GP corresponded to approximately 2.00 cm^2^. This measurement was used to calculate the unit drug loading of each compound for subsequent release, permeation, and pharmacokinetic studies. All samples were analyzed in triplicate for each batch.

### 2.6. In Vitro Dissolution Study of GP

An improved vertical Franz diffusion cell was used with a drug release area of 0.785 cm^2^ and a receiver chamber volume of 5 mL. The receptor medium consisted of physiological saline containing 30% (*v*/*v*) ethanol. A 0.8 μm pore-size microporous membrane was placed flat on the receiver chamber. The membrane was pre-soaked prior to use to ensure full hydration. Under the experimental conditions employed, the membrane was not considered to be the rate-limiting barrier for drug diffusion. The GP was cut into circular pieces matching the drug release area and closely adhered to the microporous membrane. In addition, an active pharmaceutical ingredient (API) control group was prepared and evaluated under identical Franz diffusion conditions. For the API control, pure sinomenine, osthole, cinnamaldehyde, and imperatorin were individually dissolved in the same receptor medium at concentrations corresponding to their respective unit drug loading per release area (μg·cm^−2^) in GP. A defined volume of this solution was then applied onto the diffusion membrane to match the effective release area (0.785 cm^2^) of the GP samples. All other experimental conditions were kept identical to those of the GP group. An appropriate volume of receptor medium was added to the receiver chamber up to the calibration mark, ensuring no air bubbles were present. A magnetic stir bar was placed in the receiver chamber and stirred at 600 rpm. The water bath temperature was maintained at 37 ± 0.5 °C. Samples of 5 mL receptor medium were withdrawn at 1, 2, 4, 6, 8, 10, 12, and 24 h, and an equal volume of pre-warmed receptor medium was immediately replenished to maintain sink conditions. Air bubbles in the receiver chamber were removed after sampling. After vacuum concentration, samples were reconstituted in 1 mL chromatographic grade methanol and filtered through a 0.22 μm microporous membrane prior to quantification of sinomenine, osthole, cinnamaldehyde and imperatorin [26]. For each GP batch, in vitro release experiments were performed using three parallel Franz diffusion cells. The mean values of the triplicates were used to obtain batch-level release data for subsequent inter-batch comparison and correlation analysis.

The cumulative amount of drug released per unit area (*Q_R_*, μg·cm^–2^) at the nth sampling time point was calculated according to the following equation:

(1) $$Q_{R}=\frac{V_{s}C_{n}+\sum_{i=1}^{n-1}C_{i}V}{S},$$

*V_s_* is the volume of receptor medium in the receiver chamber (5 mL); *C_n_* is the drug concentration measured at the nth sampling point (μg·mL^−1^); C_i_ is the drug concentration at the ith sampling point (μg·mL^−1^); *V* is the sample volume withdrawn (5 mL); and *S* is the effective release area of the diffusion cell (0.785 cm^2^).

*Q_R_* was calculated according to Equation (1). For graphical presentation and comparison across components, the cumulative release percentage (%) was also calculated according to Equation (2) by normalizing *Q_R_* to the total drug content (*Q_total_*, μg·cm^−2^) of each sample. IVIVC analysis was performed using *Q_R_* values to ensure consistency with in vivo concentration units.

(2) $$\mathrm{Release}\;\mathrm{percentage}(\%)=\frac{Q_{R}}{Q_{total}}\;\times \;100\%,$$

The cumulative release data were analyzed using different kinetic models to describe drug release behavior. Zero-order, first-order, and Higuchi models were applied to evaluate concentration dependence and diffusion-controlled release. In addition, the Korsmeyer–Peppas (K–P) equation was used to analyze the data up to 60% of the cumulative release fraction, and the release exponent (*n*) was employed to infer the underlying release mechanism. Model fitting was performed using linear regression of the transformed equations, and the goodness of fit was assessed by the coefficient of determination (*R*^2^) and the corrected Akaike information criterion (AICc).

### 2.7. Statistical Analysis of Structure–Release Correlation

Correlation analysis was performed at the batch level (*n* = 6), where each data point represents the average value of replicate measurements within each GP batch. Pearson product-moment correlation coefficients (Pearson’s *r*) were computed between the Higuchi release constant (*k_h_*) and each microstructural descriptor. Subsequently, ordinary least-squares (OLS) linear models with *k_h_* as the response were fitted to estimate slope coefficients (*β*_1_), 95% confidence intervals (*CI*), and *R*^2^.

### 2.8. Ex Vivo Permeation Study of GP

An improved vertical Franz diffusion cell was used with a release area of 0.785 cm^2^ and a receiver chamber volume of 5 mL. Abdominal skin was excised from rats after sacrifice, and subcutaneous fat was carefully removed without damaging the dermal layer. Before the permeation experiment, the integrity of the skin was assessed by visual inspection, and only intact skin without visible damage was used for the permeation study to ensure barrier integrity. The skin was mounted between the donor and receiver chambers with the stratum corneum facing the donor compartment. The receptor medium consisted of physiological saline containing 30% (*v*/*v*) ethanol to ensure adequate solubility of the marker compounds. The receptor chamber was filled to the calibration mark, ensuring no air bubbles were present. GP was applied onto the skin surface under finite-dose conditions over the same effective release area. The system was maintained at 37 ± 0.5 °C and stirred at 600 rpm using a magnetic stir bar. At 1, 2, 4, 6, 8, 10, 12, and 24 h, the entire 5 mL of receptor medium was withdrawn and immediately replaced with an equal volume of fresh pre-warmed receptor medium to maintain constant volume and sink conditions. Air bubbles in the receiver chamber were removed after sampling. Collected samples were evaporated under reduced pressure, reconstituted in 1 mL of LC–MS grade methanol, filtered through a 0.22 μm microporous membrane, and analyzed for sinomenine, osthole, cinnamaldehyde and imperatorin [27]. For each GP batch, permeation experiments were performed using three parallel Franz diffusion cells. The mean values of the triplicates were used to obtain batch-level permeation data for subsequent analysis.

The cumulative transdermal permeation per unit area (*Q_n_*, μg·cm^−2^) at the nth time point was calculated according to the following equation:

(3) $$Q_{n}=\frac{V_{s}C_{n}+\sum_{i=1}^{n-1}C_{i}V}{S},$$

*Q_n_* was calculated according to Equation (3). For graphical presentation and comparison across components, the cumulative permeation percentage (%) was also calculated according to Equation (4) by normalizing *Q_n_* to the *Q_total_* of each sample. IVIVC analysis was performed using *Q_n_* values to ensure consistency with in vivo concentration units.

(4) $$\mathrm{Permeation}\;\mathrm{percentage}(\%)=\frac{Q_{n}}{Q_{total}}\;\times \;100\%,$$

The cumulative permeation data were analyzed to calculate transdermal parameters. steady-state flux (*J_ss_*) was calculated according to Equation (5) as the slope of the linear portion of the cumulative permeation–time curve; The lag time (*t_lag_*) was obtained by extrapolating the steady state regression line to the x-axis; And apparent skin permeability (*P_skin_*) was calculated according to Equation (6) [28,29]. In addition, zero-order, first-order, and Higuchi models were fitted for comparison, but transdermal kinetics were primarily evaluated using flux-based parameters [30,31].

(5) $$J_{ss}=\frac{∆Q}{∆t\times A},$$

(6) $$P_{skin}=\frac{J_{ss}}{\Delta C},$$

where *J_ss_* is the steady state flux (μg·cm^−2^·h^−1^), ΔQ/Δt is the slope of the linear portion of the cumulative permeation–time curve, A is the effective release area (cm^2^), and Δ*C* represents the concentration gradient across the skin, which was approximated as the apparent donor concentration under sink conditions (μg·cm^−3^).

### 2.9. ATR–FTIR Studies of GP-Treated Skin

ATR–FTIR was employed to investigate the effects of GP on stratum corneum lipid order and keratin conformation. According to the procedures of the in vitro permeation experiment, full-thickness abdominal skin excised from rats was used. The skin was carefully prepared by removing subcutaneous fat without damaging the dermal layer.

For comparison, a placebo matrix without active pharmaceutical ingredients was prepared according to the general method for black plaster described in the Chinese Pharmacopoeia [32] and used to treat the skin.

Skin samples were divided into three groups: GP-treated skin (GP group), vehicle-treated skin (Vehicle group), and untreated skin incubated under the same conditions (CON group). Six independent skin samples were analyzed for each group (*n* = 6). The dried skin samples were mounted on the diamond ATR accessory, and the pressure head was adjusted to ensure close contact between the sample and the crystal. Spectra were recorded in the range of 400–4000 cm^−1^ with a resolution of 8 cm^−1^ [10,33].

### 2.10. DSC Studies of GP-Treated Skin

DSC was conducted on the same batch of GP-treated, vehicle-treated, and control skin samples to evaluate the effects of GP on stratum corneum lipid phase transition and keratin stability. Full-thickness abdominal skin excised from rats was carefully prepared by removing subcutaneous fat without damaging the dermal layer, following the procedures of the ex vivo permeation study.

For the vehicle-treated group, a placebo matrix without active pharmaceutical ingredients was applied to the skin (Vehicle group). Skin samples were divided into three groups: GP-treated skin (GP group), vehicle-treated skin (Vehicle group), and untreated skin (CON group) (*n* = 6). The samples were ground in a mortar under continuous liquid nitrogen cooling to prevent softening and adhesion, and then lyophilized to obtain dry skin powder. DSC was conducted from 20 to 250 °C at a heating rate of 10 °C·min^−1^ under a nitrogen purge [34].

### 2.11. In Vivo Microdialysis Studies

Twelve rabbits were randomly assigned to six GP batches. Pharmacokinetic analyses were performed at the batch level (*n* = 6).

Microdialysis probes were connected to a microdialysis pump and perfused with physiological saline containing 30% (*v*/*v*) ethanol at a constant flow rate of 2.0 μL/min for 30 min to achieve equilibration. Twelve New Zealand white rabbits were anesthetized via the marginal ear vein and fixed in the supine position. The fur over both posterior knee joints was carefully shaved to expose the application sites. Microdialysis probes were simultaneously inserted into the SC tissue and the IA cavity. The probes were continuously perfused with physiological saline containing 30% (*v*/*v*) ethanol at a flow rate of 2.0 μL/min and equilibrated in vivo for 1 hour. Approximately 2.0 g of GP (covering an area of 2.0 cm^2^) was evenly applied to the depilated knee area. Dialysate samples were collected simultaneously from both the SC and IA sites, with 120 μL collected per sample over a total period of 12 h [35]. Microdialysis sampling was performed over a 12-h observation window. Extending the sampling period would require prolonged anesthesia and stable probe positioning in rabbits, which was difficult to maintain for extended durations. Therefore, pharmacokinetic parameters were evaluated within the predefined 12 h sampling window [36,37].

After sample collection, the plaster was removed, and the administration site was gently cleaned. The perfusate was then replaced with a physiological saline containing 30% (*v*/*v*) ethanol containing known concentrations of sinomenine, osthole, cinnamaldehyde and imperatorin. Six dialysate samples (40 μL each) were collected to determine the concentrations of the analytes and calculate the in vivo relative recovery using the retrodialysis method. The relative recovery values ranged from 30% to 70%, indicating efficient probe performance. The concentrations measured in dialysates after drug administration were corrected accordingly [38,39,40,41].

Dialysate concentrations were quantified by dual-site microdialysis combined with UPLC–MS/MS. Pharmacokinetic profiles were constructed, and key pharmacokinetic parameters were calculated using Phoenix WinNonlin (version 8.3.5, Certara Inc., Princeton, NJ, USA).

### 2.12. IVIVC Analysis

To establish a quantitative IVIVC, paired datasets were constructed by aligning the sampling time points of in vitro release, ex vivo permeation, and in vivo pharmacokinetics. The *Q_R_* and *Q_n_* were used as independent variables, normalized to cumulative percentages when appropriate. Drug concentrations in SC tissue and the joint cavity obtained by microdialysis were treated as dependent variables.

Linear regression analyses were performed using Origin 2021 (OriginLab, Northampton, MA, USA), and the strength of correlation was evaluated by *R*^2^. Because the *R*^2^ values differed among sinomenine, osthole, cinnamaldehyde and imperatorin, the correlation results were interpreted separately for each marker compound. Nonlinear regression was not applied, as the focus was on assessing predictive trends rather than maximizing model fit. This analysis corresponds to a Level B IVIVC approach, as it evaluates statistical correlations between in vitro release, ex vivo permeation and in vivo pharmacokinetic profiles without establishing a point-to-point relationship.

### 2.13. Statistical Analysis

Statistical analyses were performed using SPSS (version 25.0) and Origin 2021. Data are presented as mean ± standard deviation (SD). Descriptive statistics were used to summarize quantitative data. Correlation and regression analyses (Pearson’s *r* and ordinary least-squares linear regression) were performed to evaluate structure–release relationships and IVIVC at the batch level (*n* = 6). The significance of Pearson’s *r* was evaluated using a two-sided test with a threshold of *p* < 0.05.

Because the analyses were exploratory and correlational in nature, no formal between-group hypothesis testing was performed. Sample sizes were determined according to the exploratory design, the availability of independent GP batches, routine replication practice for in vitro and ex vivo experiments, and ethical considerations for animal use.

## 3. Results

### 3.1. Microstructure of GP

**Figure 1:**
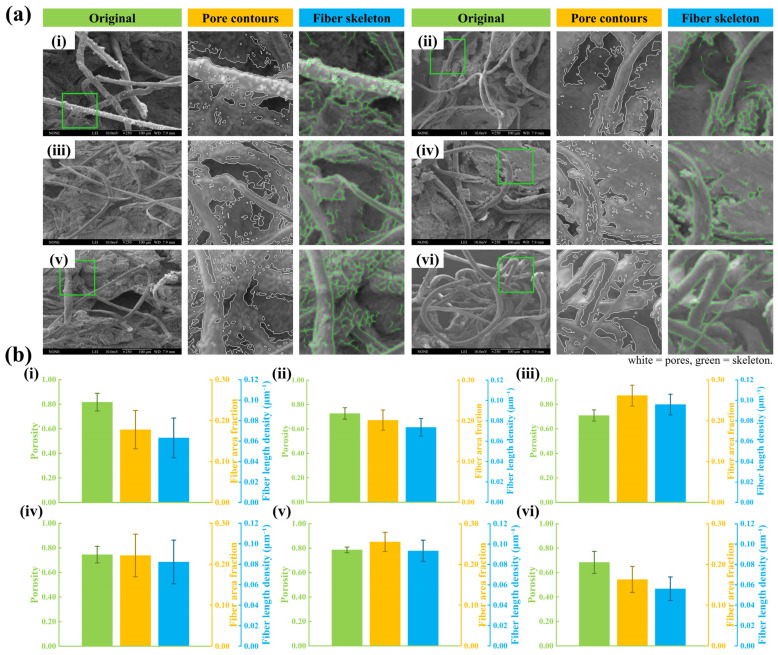
FESEM-based microstructural characterization and quantitative analysis of GP. (**a**) Representative FESEM images of six independent batches (**i**–**vi**), with pore contours (white) and skeletonized fibers (green) obtained from ROI-based image processing. (**b**) Quantification of porosity, fiber area fraction, and fiber length density across batches (**i**–**vi**). Data are presented as mean ± SD (*n* = 6). For each batch, three images were analyzed with four fixed ROIs per image.

Under FESEM, the pretreated GP exhibited a highly entangled three-dimensional fibrous network with a biphasic architecture, where fibrous structures were interspersed with an oil phase occupying the interstitial regions. For visualization, Figure 1 shows the original micrograph with pore contours and a skeletonized fiber overlay from one representative ROI quadrant within the predefined fixed ROI grid. Batch-level mean ± SD values of porosity, fiber area fraction, and fiber length density (*n* = 6 batches) are summarized in the inset bar charts and were used for subsequent correlation analysis.

Per-batch microstructural metrics are summarized in Table S5. The overall ranges were as follows: porosity 0.684–0.817, fiber area fraction 0.163–0.262, and fiber length density 0.056–0.096 μm^−1^.

### 3.2. Quantitative Analysis of Active Components in GP

The contents of sinomenine, osthole, cinnamaldehyde, and imperatorin in GP were quantified using a validated UPLC–MS/MS method. As shown in Table 2, the mean contents (μg·g^−1^) were 0.26 ± 0.04, 8.58 ± 0.23, 8.92 ± 0.21, and 15.26 ± 0.32, respectively (*n* = 6). Each batch was analyzed in triplicate, and the average values were used for inter-batch comparison. The relatively small standard deviations across the six batches indicate limited variability among batches.

Based on the experimentally determined plaster-area ratio, these contents correspond to unit loadings of approximately 0.26 μg·cm^−2^ for sinomenine, 8.58 μg·cm^−2^ for osthole, 8.92 μg·cm^−2^ for cinnamaldehyde, and 15.26 μg·cm^−2^ for imperatorin. These values were used as reference amounts in the subsequent in vitro release, ex vivo permeation, and in vivo pharmacokinetic experiments.

### 3.3. In Vitro Drug Release Studies

**Figure 2:**
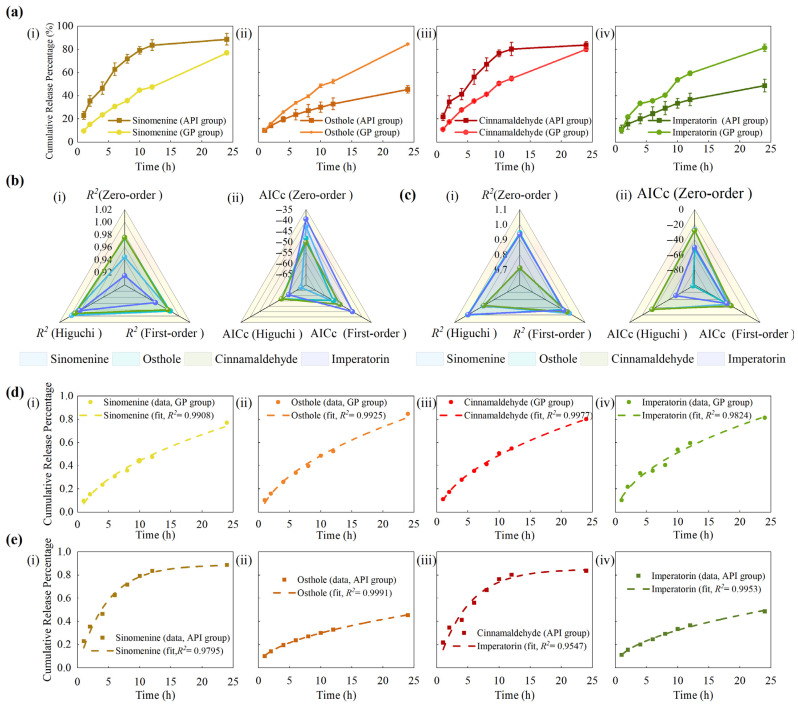
In vitro drug-release behaviors and kinetic model fitting of four marker compounds from GP and API groups. (**a**) Cumulative release profiles of (**i**) sinomenine, (**ii**) osthole, (**iii**) cinnamaldehyde, and (**iv**) imperatorin over 24 h for GP and API groups. (**b**) Comparison of *R*^2^ and AICc values among the zero-order, first-order, and Higuchi models for the GP group. (**c**) Comparison of *R*^2^ and AICc values among the zero-order, first-order, and Higuchi models for the API group. (**d**) Representative Higuchi-fitted curves for the GP group (best-fit model): (i) sinomenine, (**ii**) osthole, (**iii**) cinnamaldehyde, and (**iv**) imperatorin. (**e**) Representative best-fit curves for the API group: (**i**) sinomenine and (**iii**) cinnamaldehyde followed the first-order model, whereas (**ii**) osthole and (**iv**) imperatorin followed the Higuchi model. Data are presented as mean ± SD (*n* = 6 batches). For each batch, release profiles were obtained from three parallel Franz diffusion cells and averaged prior to analysis.

Cumulative release of the four marker compounds from GP was evaluated using a modified Franz diffusion cell. As shown in Figure 2a, all compounds exhibited sustained release over 24 h, with cumulative percentages of 76.94 ± 1.68% for sinomenine, 84.58 ± 0.73% for osthole, 80.10 ± 2.06% for cinnamaldehyde, and 81.35 ± 3.25% for imperatorin. In the API control group, the cumulative release at 24 h reached 88.67 ± 4.97% for sinomenine, 45.50 ± 3.24% for osthole, 83.72 ± 2.83% for cinnamaldehyde, and 48.71 ± 5.46% for imperatorin. Corresponding cumulative release data for both groups are provided in Table S6. These values represent mean ± SD calculated across six GP batches (*n* = 6), where each batch value was obtained by averaging three parallel Franz diffusion cells.

To further characterize the release profiles, the cumulative release data of both groups were fitted to three classical kinetic models: zero-order, first-order, and Higuchi. For the GP group, the Higuchi model consistently provided the best fit, as shown in Figure 2b, with *R*^2^ values ranging from 0.9908 to 0.9977 and AICc values between −56.87 and −67.46, clearly outperforming the other two models. The corresponding fitting curves are shown in Figure 2d. In contrast, the API group exhibited compound-dependent behavior, as illustrated in Figure 2c: sinomenine and cinnamaldehyde were best described by the first-order model, with *R*^2^ values of 0.9795 and 0.9612 and AICc values of −48.6 and −44.14, respectively, whereas osthole and imperatorin followed the Higuchi model, showing *R*^2^ values of 0.9998 and 0.9953 and AICc values of −97.32 and −71.22. The corresponding fitting curves are shown in Figure 2e and detailed kinetic parameters are summarized in Table S7. These results indicate differences in release behavior between the GP and API groups. The fitted kinetic models suggest that the release profiles differ between the GP and API groups, in accordance with theoretical descriptions of drug release from polymeric and matrix-based systems [42,43,44,45]. These results are consistent with the sustained release profiles observed in Figure 2a.

**Figure 3:**
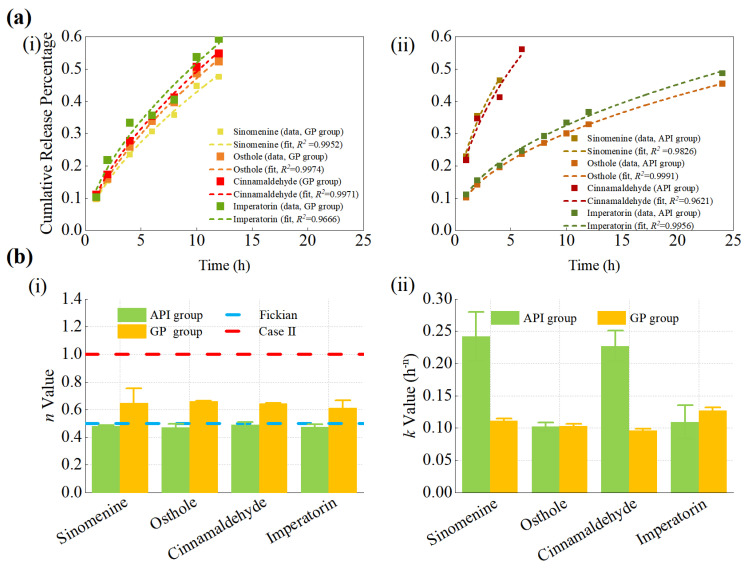
K–P model analysis of drug release from GP and API groups. (**a**) K–P fittings within the initial 60% release region for both groups. (**b**) Comparison of K–P parameters between the GP and API groups: (**i**) *n* values and (**ii**) *k* values. Data are presented as mean ± SD (*n* = 6 batches).

To further characterize the early-stage kinetics, the initial 60% of the cumulative release profiles were fitted using the K–P model [46,47], and the fitted curves are shown in Figure 3a. As illustrated in Figure 3b, the GP group exhibited K–P release constants (*k*) ranging from 0.0962 to 0.1269 h^−n^ and *n* between 0.61 and 0.66, indicating a non-Fickian anomalous diffusion mechanism involving both diffusion and matrix relaxation. In contrast, the API group exhibited higher *k* values, ranging from 0.1020 to 0.2421 h^−n^, while the *n* values were between 0.47 and 0.49, corresponding to Fickian or near-Fickian diffusion. The detailed kinetic parameters are summarized in Table S8. These results indicate differences in release behavior between the GP and the structure-free control, consistent with its sustained-release characteristics.

### 3.4. Microstructure–Release Correlation Studies

**Figure 4:**
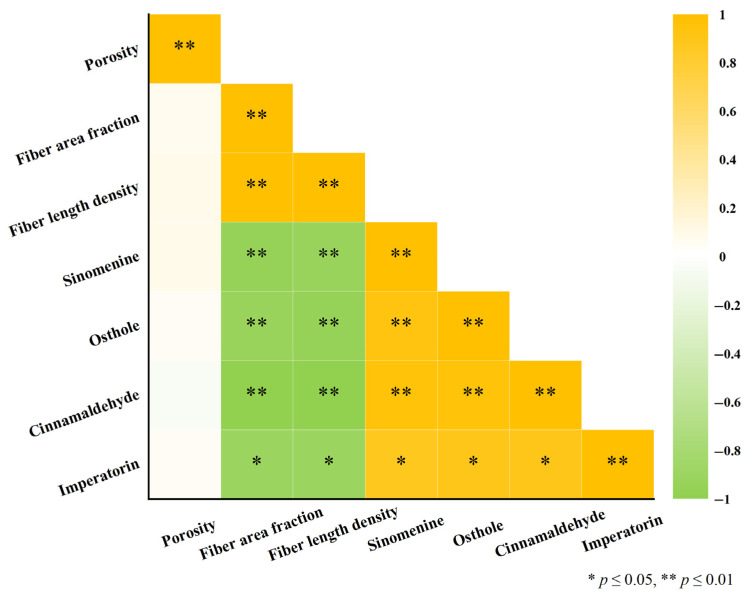
Microstructure–release correlation matrix of GP. Correlation matrix among fiber area fraction, fiber length density, porosity, and the *k_h_* of four marker compounds. Correlation analysis was performed at the batch level using six independent GP batches (*n* = 6), where each data point represents the average value of replicate measurements within each batch. Statistical significance of Pearson’s correlation coefficients was evaluated (* *p* < 0.05, ** *p* ≤ 0.01).

**Figure 5:**
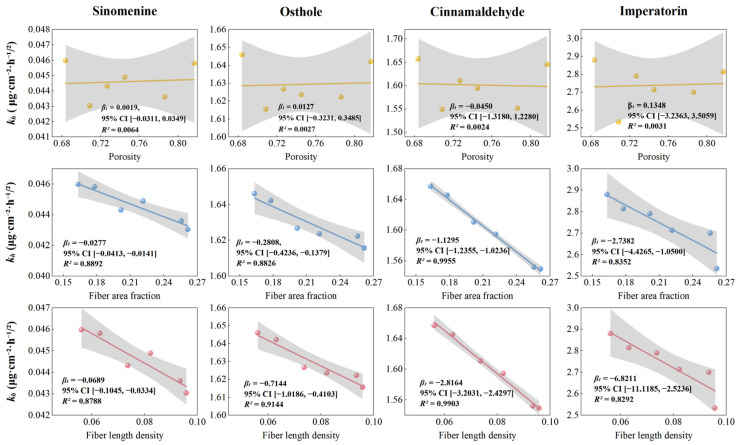
Linear regression analysis between microstructural parameters and release kinetics of GP. Linear regression plots showing relationships between the *k_h_* and structural parameters (porosity, fiber area fraction, and fiber length density) for sinomenine, osthole, cinnamaldehyde, and imperatorin. Each data point represents one batch (*n* = 6).

Correlation analysis was performed across six independent GP batches (*n* = 6). As shown in Figure 4 and Figure 5, fiber area fraction exhibited a negative correlation with *k_h_* across all marker compounds, with *r* values ranging from −0.9139 to −0.9977. A similar negative association was observed for fiber length density, whereas porosity showed minimal correlation with *k_h_*. These results indicate that batch-level variations in the quantified structural descriptors are statistically associated with variations in the *k_h_*. Specifically, higher fiber area fraction and greater fiber length density coincided with lower *k_h_* values. It should be noted that these correlations were derived from a limited number of batches (*n* = 6) and therefore represent associative trends rather than definitive mechanistic proof. Nevertheless, a consistent directionality was observed across all compounds.

### 3.5. Ex Vivo Skin Permeation Studies

**Figure 6:**
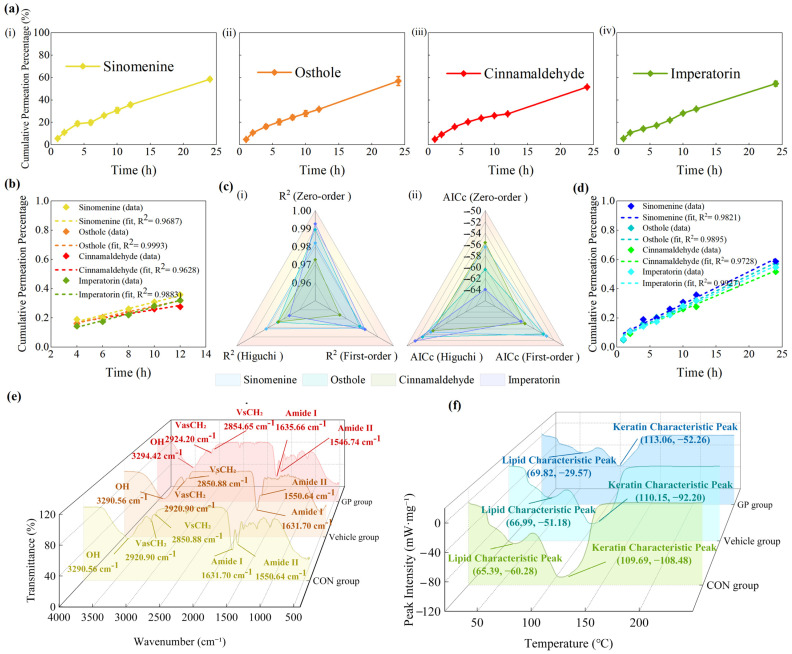
Ex vivo skin permeation characteristics and structural analyses of GP-treated skin. (**a**) Cumulative ex vivo permeation profiles of (**i**) sinomenine, (**ii**) osthole, (**iii**) cinnamaldehyde, and (**iv**) imperatorin over 24 h. (**b**) Linear regression fittings of the 4–12 h interval for the four marker compounds. (**c**) Comparison of (**i**) *R*^2^ and (**ii**) AICc values among the zero-order, first-order, and Higuchi models. (**d**) Zero-order kinetic fittings of cumulative permeation profiles. (**e**) ATR–FTIR spectra of CON, Vehicle, and GP groups showing lipid and protein band shifts. (**f**) DSC thermograms of CON, Vehicle, and GP groups illustrating thermal transition changes in lipid and keratin domains. Data are presented as mean ± SD (*n* = 6).

The ex vivo skin permeation of sinomenine, osthole, cinnamaldehyde, and imperatorin from GP was evaluated using the Franz diffusion cell. As shown in Figure 6a, all four compounds exhibited a sustained, time-dependent increase in permeation over 24 h, and the corresponding *Q_n_* at each sampling point are provided in Table S9. Linear regression of the 4–12 h interval in Figure 6b, which represented the most linear portion of the permeation profiles and minimized the influence of the initial lag phase and the terminal plateau, yielded high correlation coefficients with *R*^2^ values ranging from 0.9628 to 0.9993, and was used for the calculation of permeation parameters, as summarized in Table 3. The resulting *J_ss_* ranged from 0.0058 to 0.3524 μg·cm^−2^·h^−1^, with imperatorin showing the highest flux and sinomenine the lowest. Back extrapolation of the regression lines suggested negligible lag phases (*t_lag_* ≈ 0 h). For the estimation of *P_skin_*, the experimentally determined contents of the marker compounds in GP were adopted as apparent donor concentrations, which under sink conditions were used to approximate *ΔC*, as shown in Table 3. This approach is consistent with practices commonly applied in in vitro permeation test (IVPT) studies of semisolid formulations, where explicit donor concentrations are not directly measurable [48,49,50,51]. Under this assumption, the calculated *P_skin_* values corresponded to the regression slopes and ranged from 0.0144 to 0.0231 cm·h^−1^. The calculated permeation parameters further supported these findings: *P_skin_* values fell within 0.0144–0.0231 cm·h^−1^, indicating broadly comparable normalized permeation potentials. Compared with the in vitro release results, the ex vivo permeation of GP marker compounds was markedly lower, suggesting that transdermal permeation is more limited than drug release under the present experimental conditions.

To further characterize the permeation kinetics, the cumulative permeation data were fitted to three classical models: zero-order, first-order, and Higuchi. The comparative fitting outcomes are presented in Figure 6c and detailed regression values are summarized in Table S10. Across all four compounds, the zero-order model consistently yielded the lowest AICc values, ranging from −55.67 to −63.97, together with the highest *R*^2^ values of 0.9728–0.9927, outperforming both the first-order and Higuchi models. Representative fitted curves are shown in Figure 6d. These results indicate that the transdermal permeation of sinomenine, osthole, cinnamaldehyde, and imperatorin from GP is best described by zero-order kinetics, suggesting a relatively constant permeation rate over the experimental duration. This kinetic pattern is consistent with a relatively constant permeation rate during the experimental period [52]. Permeation parameters were calculated at the batch level (*n* = 6), where each value represents the average of three parallel Franz diffusion cells per batch.

### 3.6. ATR–FTIR Analysis of GP-Treated Skin

The ATR–FTIR spectra of CON, Vehicle, and GP groups are shown in Figure 6e, and the corresponding peak positions are summarized in Table S11. In the lipid region, the symmetric (νsCH_2_, ~2850 cm^−1^) and asymmetric (νasCH_2_, ~2920 cm^−1^) CH_2_ stretching vibrations were clearly observed [53,54].

Compared with the CON group, the Vehicle group exhibited slight shifts in these peaks to higher wavenumbers, with νasCH_2_ shifting from 2920.90 to 2921.28 cm^−1^ and νsCH_2_ from 2850.88 to 2851.37 cm^−1^, indicating a modest reduction in lipid chain order. Notably, the GP group showed more pronounced shifts, with νasCH_2_ reaching 2924.20 cm^−1^ and νsCH_2_ reaching 2854.65 cm^−1^, suggesting a greater disruption of lipid packing and increased fluidity of the stratum corneum [55,56]. In the protein region, characteristic Amide I (~1650 cm^−1^) and Amide II (~1540 cm^−1^) bands were identified [57]. The Vehicle group exhibited minor changes in peak positions, with Amide I shifting from 1631.70 to 1632.56 cm^−1^ and Amide II from 1550.64 to 1549.03 cm^−1^, whereas the GP group induced a moderate upshift of Amide I to 1635.66 cm^−1^ and a downshift of Amide II from 1550.64 to 1546.74 cm^−1^, suggesting conformational rearrangements in keratin [58].

### 3.7. DSC Analysis of GP-Treated Skin

The DSC thermograms of the CON, Vehicle, and GP groups are shown in Figure 6f, with the corresponding peak temperatures and enthalpy values summarized in Table S12. As generally reported, the cooperative phase transition of intercellular lipids occurs within approximately 35–90 °C, whereas keratin transitions are observed around 90–120 °C [34]. In the CON group, the lipid transition exhibited a characteristic peak temperature (*T_peak_*) of 65.37 °C and a corresponding peak intensity of −60.28 mW·mg^−1^. The Vehicle group showed a slight increase in transition temperature and a reduction in peak intensity, indicating partial disruption of lipid organization. In contrast, the GP group exhibited a more pronounced increase in lipid transition temperature to 69.82 °C and a marked decrease in peak intensity to −29.57 mW·mg^−1^, suggesting significant alterations in lipid packing and thermal behavior [59].

For the protein domain, the keratin-related endotherm appeared at 109.69 °C in the CON group. The Vehicle group exhibited moderate changes, whereas the GP group showed a clearer shift to 113.06 °C, accompanied by a reduction in peak intensity from −108.48 to −52.56 mW·mg^−1^, indicating alterations in the thermal transition behavior of keratin proteins [60,61]. Overall, these DSC findings indicate that both the matrix and active components of GP influence the thermal properties of stratum corneum lipids and proteins, with GP producing more pronounced effects.

### 3.8. In Vivo Synchronous Microdialysis in SC and IA Compartments

**Figure 7:**
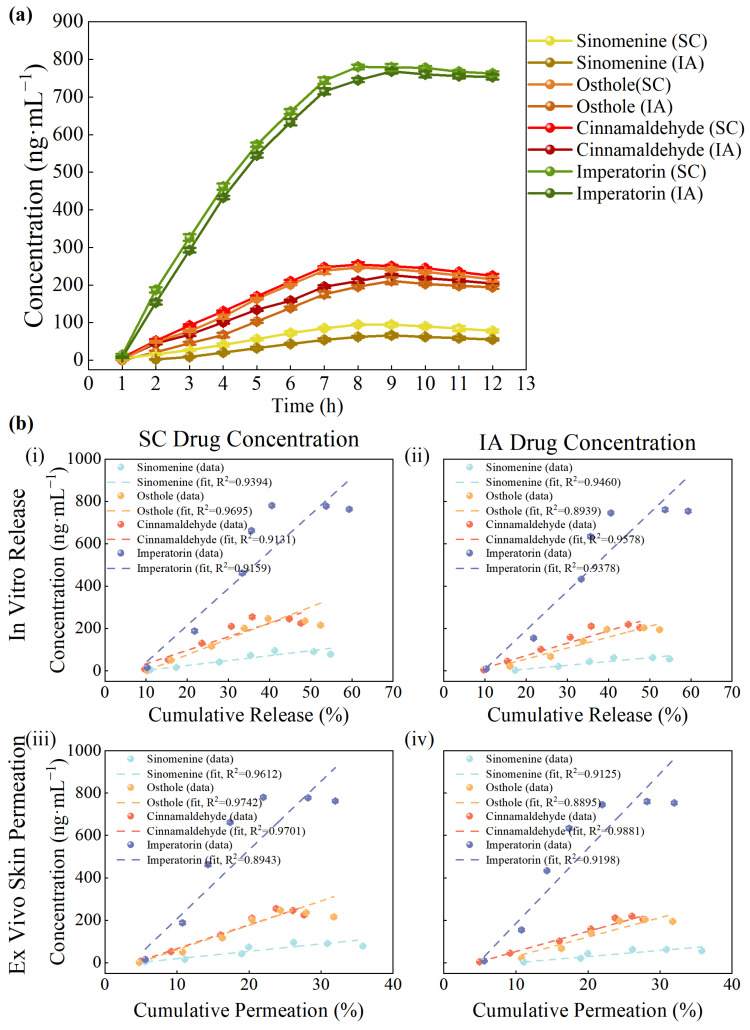
In vivo microdialysis pharmacokinetics and IVIVC analysis of GP. (**a**) Concentration–time profiles of sinomenine, osthole, cinnamaldehyde, and imperatorin in SC tissue and IA cavity (*n* = 6 rabbits). (**b**) Linear correlations: (**i**) In vitro cumulative release versus SC concentrations; (**ii**) In vitro cumulative release vs. IA concentrations; (**iii**) Ex vivo skin permeation vs. SC concentrations; (**iv**) Ex vivo skin permeation vs. IA concentrations. IVIVC analysis was performed using six GP batches (*n* = 6).

Figure 7a illustrates the concentration–time profiles of sinomenine, osthole, cinnamaldehyde, and imperatorin in both SC tissue and the IA cavity, with the raw data provided in Table S13. All four compounds exhibited a time-dependent increase followed by a plateau phase. The SC *T_max_* values were generally observed at 7–8 h, whereas the IA cavity *T_max_* was slightly delayed to 8–9 h. Overall, the concentration–time curves in the two compartments showed comparable trends, suggesting similar temporal profiles. *T_max_* values ranged from approximately 7 to 9 h across the four compounds. After reaching peak levels, concentrations exhibited a plateau or declining trend within the 12 h observation window, indicating that the major exposure phase was captured within this period.

The pharmacokinetic parameters are summarized in Table 4. Among the four compounds, imperatorin exhibited the greatest local exposure, with *AUC*_0–12_ values of 6538 and 6452 ng·h·mL^−1^ in the SC and IA compartments, respectively, and a Cmax of approximately 780 ng·mL^−1^, which is consistent with its measurable transdermal permeation and tissue exposure observed in this study. Cinnamaldehyde ranked next, with *AUC*_0–12_ values of 1103 and 1066 ng·h·mL^−1^ and a Cmax of about 126–129 ng·mL^−1^, showing nearly identical exposure between the two sites. By contrast, osthole and sinomenine showed lower Cmax and *AUC* values, and their IA exposure was moderately reduced relative to SC levels, as reflected by the IA/SC *AUC* ratio [62], which was about 0.82–0.85. *MRT*_0–12_ values were consistently between 7 and 8 h in both compartments, with minimal differences, consistent with the gradual rise and plateau observed in the concentration–time profiles. Overall, these findings reveal distinct exposure characteristics among the compounds: imperatorin and cinnamaldehyde achieved balanced delivery across compartments, whereas osthole and sinomenine displayed relatively limited IA penetration.

### 3.9. IVIVC Results

As shown in Figure 7b(i,ii), cumulative drug release exhibited linear correlations with in vivo concentrations in both compartments. For SC tissue, *R*^2^ were 0.9394 for sinomenine, 0.9695 for osthole, 0.9131 for cinnamaldehyde, and 0.9159 for imperatorin. For the IA cavity, the *R*^2^ values were 0.9460, 0.8939, 0.9578, and 0.9378, respectively. These values indicate an apparent association between in vitro release profiles and in vivo exposure levels, with IA cavity correlations being slightly stronger overall. Detailed linear regression parameters, including slope and *R*^2^ values for each compound, are summarized in Table S14.

As shown in Figure 7b(iii,iv), ex vivo skin permeation also showed correlations with in vivo concentrations, although with greater variability across compounds. The *R*^2^ values for SC tissue concentrations were 0.9612 for sinomenine, 0.9742 for osthole, 0.9742 for cinnamaldehyde, and 0.8943 for imperatorin. For the IA cavity, the values were 0.9125, 0.8895, 0.9881, and 0.9198, respectively. These results indicate that permeation data are associated with in vivo concentration profiles, though the strength of correlation differed among compounds.

Overall, both in vitro release and ex vivo skin permeation showed associations with in vivo pharmacokinetic profiles, with release data generally exhibiting slightly higher and more consistent correlations. These findings support the presence of a Level B IVIVC [63] relationship for GP under the present experimental conditions. However, the observed correlations should be interpreted as descriptive rather than predictive, considering the complexity of the GP matrix and the limitations of the current experimental design.

## 4. Discussion

### 4.1. Microstructure and Release Behavior

FESEM revealed that GP exhibits a biphasic fibrous–oil architecture composed of a densely interlaced fibrous scaffold interspersed with oil domains [42,43,64,65]. Compared with the API control, which lacked this organized matrix, the GP formulation showed different kinetic behaviors. In the control group, sinomenine and cinnamaldehyde followed first-order kinetics, whereas osthole and imperatorin were better fitted by the Higuchi model. Further analysis using the K–P model for the initial 60% release yielded *n* values of approximately 0.61–0.66, indicating non-Fickian release behavior. Further interpretation of the kinetic models provides insight into the release mechanism. The Higuchi model describes drug release from a matrix system assuming a diffusion-controlled process under sink conditions, where drug molecules diffuse through a porous matrix while the matrix remains relatively intact. The K–P model is a semi-empirical model used to describe drug release from polymeric systems, and the *n* value provides insight into the release mechanism. In general, *n* ≤ 0.45 indicates Fickian diffusion, 0.45 < *n* < 0.89 indicates anomalous transport, and *n* ≥ 0.89 indicates case-II transport.

**Figure 8:**
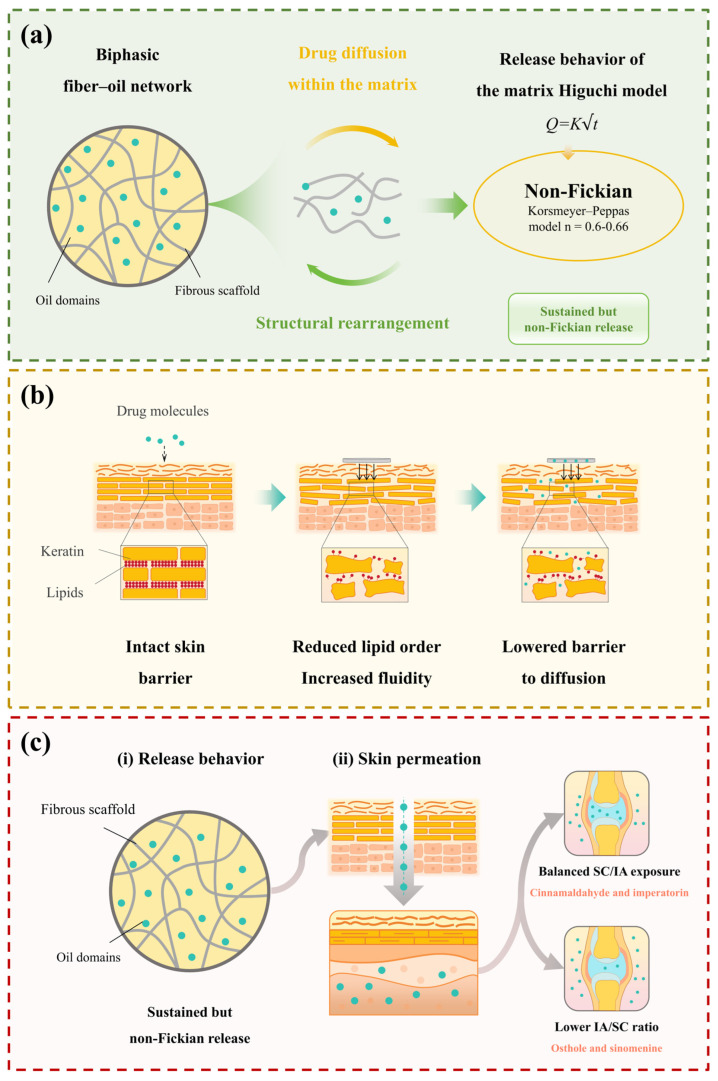
Conceptual illustration summarizing the relationships among GP microstructure, in vitro release behavior, skin permeation, and local drug exposure. (**a**) Schematic representation of the biphasic fibrous–oil microstructure observed in GP and its association with diffusion-dominated drug release behavior in vitro. The Higuchi and K–P model fittings indicate sustained but non-Fickian release characteristics. (**b**) Changes in the stratum corneum structure after GP treatment, as suggested by ATR–FTIR and DSC analyses, including reduced lipid order and increased membrane fluidity, which may lower the effective diffusion barrier. (**c**) Conceptual summary linking in vitro drug release, ex vivo skin permeation, and local drug exposure in SC tissue and the IA cavity.

In this study, the model fitting results and the observed *n* values suggest that drug release from GP exhibits diffusion-dominated characteristics, with possible contributions from matrix structural effects. This observation is consistent with the fibrous porous microstructure observed in the GP matrix, which may increase diffusion path length and tortuosity. However, this should be regarded as a possible mechanistic explanation rather than a definitive mechanistic conclusion. Correlation analysis between microstructural descriptors and kinetic parameters further revealed strong negative correlations between fiber area fraction and fiber length density with *k_h_* (*r* = −0.91 to −0.99), whereas porosity showed minimal association. Taken together, these findings suggest that variations in the fibrous microstructure are associated with differences in release behavior, and that the release profiles of GP are consistent with broadly consistent with diffusion-dominated release commonly reported for matrix-based delivery systems [66,67,68]. These observations should be interpreted as indicative of structure–function correlations rather than definitive mechanistic evidence. A conceptual illustration summarizing these observations is presented in Figure 8a.

### 4.2. Skin Permeation Characteristics

Fluxes were reliably estimated, providing a basis for further interpretation. Best fits to zero-order across the full 24 h window indicate that apparent permeation proceeded under an approximately constant driving force, consistent with passive diffusion limited by the skin barrier rather than by donor depletion [69]. The negligible lag time (*t_lag_* ≈ 0 h) implies that drug molecules rapidly partition into the stratum corneum, establishing a concentration gradient without a detectable delay [48].

Rank-ordering of permeation parameters highlights physicochemical influences: imperatorin, the most lipophilic, exhibited the highest *J_ss_*, whereas the polar sinomenine showed the lowest, reflecting their differential affinity for stratum corneum lipids. Cinnamaldehyde and osthole displayed intermediate values, consistent with their moderate hydrophobicity [28,70].

Spectroscopic and thermal analyses corroborate this barrier modulation. ATR–FTIR spectra indicated a shift in CH_2_ stretching bands to higher wavenumbers, consistent with reduced lipid chain order and increased fluidity; DSC revealed a lipid transition with reduced enthalpy (peak magnitude decreased) and a shifted peak temperature with altered cooperativity [71]. As illustrated in Figure 8b, these changes suggest that GP treatment may alter stratum corneum lipid organization and the keratin environment, lowering the effective barrier to diffusion without eliminating it—hence the still modest absolute fluxes compared with in vitro release. The placebo matrix also induced measurable changes in stratum corneum lipid and protein organization, indicating that the matrix itself contributed to skin-barrier modulation. However, the GP-treated group exhibited more pronounced alterations, suggesting a combined effect of the matrix and the incorporated active compounds.

### 4.3. IVIVC Relationships

Level B IVIVC showed strong linear associations between *Q_R_* and in vivo concentrations in both SC and IA compartments (*R*^2^ > 0.90), indicating that release testing may provide useful information for understanding local tissue exposure for GP under the studied conditions [72,73]. *Q_n_* also showed good correlations with in vivo levels, though with greater intercompound variability. These findings suggest that drug delivery from GP may involve two sequential processes: (i) release from the matrix and (ii) permeation across the skin barrier. The results indicate that drug availability at the skin surface is related to the release behavior of the GP matrix, whereas subsequent entry into SC and IA tissues is influenced by skin permeation processes, as illustrated in Figure 8c. It should be emphasized that these mechanistic inferences are speculative and based on observed trends; the IVIVC results primarily indicate statistical associations rather than causality.

Because Level B correlation summarizes profiles rather than enforcing point-to-point deconvolution, it captures predictive trends but should not be overinterpreted as a surrogate for Level A links.

Notably, compounds differed in how well permeation predicted tissue levels. Imperatorin and cinnamaldehyde exhibited nearly equivalent exposure between SC and IA compartments, with IA/SC *AUC*_0_–_12_ values of approximately 0.99 and 0.97, respectively, whereas osthole and sinomenine showed lower ratios of about 0.85 and 0.82. These differences reflect their distinct physicochemical and partitioning characteristics: while lipophilicity largely governs initial skin uptake, subsequent tissue distribution appears influenced by polarity, protein binding, and local clearance. Together, these findings indicate that compound-dependent factors jointly determine compartmental exposure, even when in vitro release profiles remain comparable, as illustrated in Figure 8c [74,75].

### 4.4. Methodological Considerations

In this study, physiological saline containing 30% (*v*/*v*) ethanol was used in both the in vitro release and ex vivo permeation experiments to ensure adequate solubility of the marker compounds and to maintain sink conditions throughout the experiments. The four representative marker compounds selected in this study (sinomenine, osthole, cinnamaldehyde, and imperatorin) differ substantially in their physicochemical properties and lipophilicity, and some of them exhibit limited aqueous solubility. If a purely aqueous receptor medium were used, drug concentrations in the receptor phase could approach saturation, leading to a reduced concentration gradient and a potential underestimation of the actual release or permeation capacity. Therefore, hydroalcoholic receptor media are commonly used in Franz diffusion cell studies for poorly water-soluble compounds to maintain a constant concentration gradient and to avoid solubility-limited transport [76]. Accordingly, the release and permeation results obtained in this study should be interpreted as reflecting the intrinsic release and permeation characteristics of the GP matrix under sink conditions rather than absolute permeation behavior under physiological conditions.

In addition, the temperature of the Franz diffusion system was maintained at 37 °C. Although the average skin surface temperature is approximately 32 °C, GP is a high-viscosity semisolid matrix, and lower temperatures may reduce molecular mobility within the matrix and slow drug diffusion [77,78]. Maintaining the system at 37 °C is a commonly used condition in vitro diffusion studies, which facilitates consistent comparison of release behavior under controlled conditions [79]. Moreover, the receptor compartment of the Franz diffusion cell is generally considered to represent viable epidermal and dermal tissues, whose temperature is closer to physiological temperature.

Furthermore, different animal models were used for the ex vivo permeation and in vivo microdialysis studies due to methodological considerations. Rat skin is widely used in Franz diffusion cell experiments and is considered a standard model for ex vivo permeation studies [16,76], whereas rabbits were selected for the in vivo microdialysis study because their larger joint cavity and subcutaneous tissue volume allow for microdialysis probe implantation and continuous sampling of local drug concentrations.

Overall, the in vitro release and ex vivo permeation experiments in this study were primarily designed as comparative performance tests to characterize drug release and permeation behavior from the GP matrix rather than to directly predict absolute in vivo permeation.

These methodological considerations should be taken into account when interpreting the results and extrapolating to clinical conditions.

### 4.5. Implications for GP Drug Delivery

**Figure 9:**
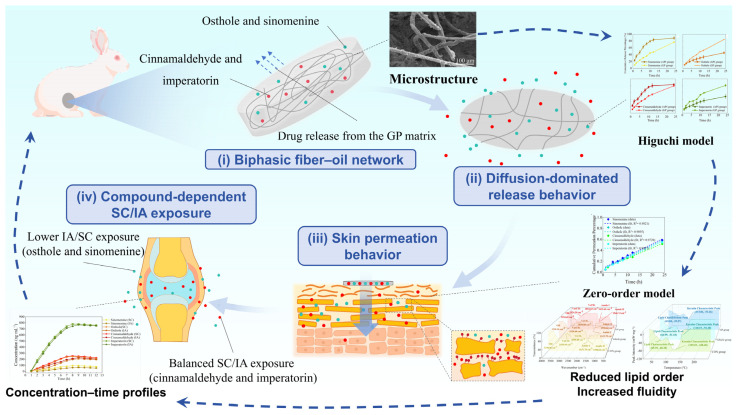
Conceptual relationships among microstructure, release behavior, skin permeation, and in vivo tissue exposure of GP: The schematic summarizes the experimental observations of this study. The biphasic fibrous–oil microstructure of GP is associated with diffusion-dominated release behavior described by Higuchi and K-P model fitting. Subsequent drug transport across the skin is consistent with passive permeation behavior, as supported by ex vivo permeation experiments and ATR–FTIR/DSC analyses. Differences in physicochemical properties among the marker compounds are associated with variations in drug exposure between SC tissue and the IA cavity.

Taken together, these findings provide an integrated view of the relationships among microstructure, drug release, skin permeation, and local tissue exposure in GP, as illustrated in Figure 9. The results suggest that transdermal delivery from GP may be associated with both microstructure-related release behavior and interactions with the skin barrier. The observed correlations indicate that in vitro release may reflect drug availability at the skin surface, while subsequent distribution into subcutaneous and intra-articular compartments may be related to permeation processes across the skin. However, these relationships should be interpreted as indicative of structure–function correlations rather than definitive mechanistic conclusions. Further studies incorporating placebo-controlled designs and expanded in vivo datasets would help to better delineate these processes and strengthen mechanistic understanding.

## 5. Conclusions

This study established an integrated framework to characterize the transdermal delivery behavior of GP by linking microstructure, release, permeation, and pharmacokinetics.

The results suggest that the fibrous–oil matrix structure is associated with sustained, diffusion-dominated drug release, consistent with Higuchi model fitting and K–P release exponents (*n* = 0.61–0.66). Transdermal permeation exhibited a slower and more stable transport profile, consistent with zero-order kinetics under the experimental conditions. Together with in vivo microdialysis findings, these results indicate that local tissue exposure may be associated with both in vitro release behavior and transdermal permeation, with additional compound-dependent differences in tissue distribution. IVIVC analysis further revealed a positive relationship between in vitro release parameters and in vivo local exposure. However, this relationship is descriptive rather than predictive, reflecting a trend-level association under the current experimental conditions.

Overall, these findings highlight structure–function correlations in GP and provide an experimental basis for the systematic evaluation of complex traditional black plaster systems.

## 6. Limitations and Future Perspectives

While this study establishes an integrated framework linking microstructure, drug release, skin permeation, and local pharmacokinetics of GP, several limitations should be acknowledged.

First, in the microstructural analysis, solvent extraction pretreatment was used prior to SEM observation to selectively remove the oil phase and expose the fibrous network of the plaster matrix. Therefore, the observed microstructure mainly represents the structural framework rather than the intact native morphology of the oil–fiber composite system. The solvent extraction process may alter the original microstructure to some extent; thus, the quantitative structural descriptors obtained from SEM should be interpreted as structural parameters for correlation analysis rather than direct representations of drug diffusion pathways within the native matrix.

Second, placebo GP was not included in the ex vivo permeation and in vivo microdialysis studies. Therefore, the specific contribution of the matrix itself to transdermal permeation and local drug exposure could not be fully separated from the effects of the active compounds, which may limit the quantitative interpretation of matrix-related effects. However, placebo-matrix-treated skin was characterized using ATR–FTIR and DSC to evaluate the influence of the matrix on the stratum corneum, providing indirect evidence for matrix-related barrier modulation.

Third, the correlation analysis between microstructural parameters and release behavior was based on six GP batches, and the relatively small sample size may increase the risk of overestimating correlation strength. In addition, some microstructural descriptors may not be fully independent, and their combined effects on drug release and permeation may be interrelated. Due to the limited sample size, multivariate analysis was not performed in order to avoid model overfitting. Therefore, the structure–function relationship described in this study should be interpreted as an association rather than a definitive mechanistic model.

In addition, the ex vivo skin model lacks the physiological complexity of living tissue, such as vascular perfusion, metabolism, and immune interactions, which may influence in vivo drug absorption and distribution. Moreover, the microdialysis sampling focused only on two anatomical sites (subcutaneous and intra-articular compartments), which limits the spatial resolution of local pharmacokinetics.

Finally, although correlations between in vitro release parameters and in vivo local exposure were observed, the current IVIVC should be considered descriptive rather than predictive. This is because transdermal drug delivery from GP is governed by both drug release from the matrix and skin barrier permeation, resulting in a complex relationship between in vitro release and in vivo exposure. In addition, the analysis was based on a limited number of batches (*n* = 6), and no point-to-point deconvolution was performed; therefore, the present results reflect a trend-level association rather than a quantitative predictive IVIVC [80].

Future studies should focus on controlled structural modification of the GP matrix and expanded sample sizes to further validate the structure–function relationship [81]. Overall, this study proposes an integrated evaluation framework for complex transdermal matrix systems, and the results should be interpreted as structure–function correlations rather than a definitive mechanistic model.

## Figures and Tables

**Table 1:** Mass spectrometry parameters for the four analytes.

Analytes	Ion Mode	Quantitative Ion Pair (*m*/*z*)	Capillary Voltage (kV)	Cone Voltage (V)	Desolvation Temperature (°C)	Collision Energy (V)
Sinomenine	ESI^+^	330.0/181.2	3.00	40	500	25
Osthole	ESI^+^	244.9/189.0	2.72	43	350	3
Cinnamaldehyde	ESI^+^	133.2/115.1	3.00	40	450	9
Imperatorin	ESI^+^	271.1/203.1	2.95	38	500	10

**Table 2:** Content of marker compounds in GP (μg·g^−1^, *n* = 6 batches).

Batch	Sinomenine	Osthole	Cinnamaldehyde	Imperatorin
1	0.22	8.41	8.83	15.37
2	0.20	8.91	8.98	15.29
3	0.29	8.38	9.07	15.29
4	0.26	8.82	9.07	15.76
5	0.29	8.58	9.03	14.85
6	0.28	8.38	8.54	15.00
Mean ± SD	0.26 ± 0.04	8.58 ± 0.23	8.92 ± 0.21	15.26 ± 0.32

Values represent mean ± SD of six batches. Each batch was analyzed in triplicate, and the mean of the triplicates was used for inter-batch comparison.

**Table 3:** Ex vivo skin permeation parameters of marker compounds from GP (*n* = 6 batches).

Compound	*J_ss_* (μg·cm^−2^·h^−1^)	*t_lag_* (h)	*P_skin_* (cm·h^−1^)	*R* ^2^
Sinomenine	0.0058 ± 0.0011	≈0	0.0223 ± 0.0043	0.9687 ± 0.0822
Osthole	0.1648 ± 0.008	≈0	0.0192 ± 0.0009	0.9993 ± 0.0265
Cinnamaldehyde	0.1288 ± 0.0027	≈0	0.0144 ± 0.0003	0.9628 ± 0.0196
Imperatorin	0.3524 ± 0.0444	≈0	0.0231 ± 0.0029	0.9883 ± 0.0392

Negative *t_lag_* values were treated as zero (no discernible lag).

**Table 4:** Pharmacokinetic parameters of the four compounds in SC and IA (*n* = 6 batches).

Compound	Type	*C_max_* (ng·mL^−1^)	*T_max_* (h)	*AUC*_0–12_ (ng·h·mL^−1^)	*MRT*_0–12_ (h)
Sinomenine	SC	42.82	8	357.95	7.8
	IA	34.59	9	293.68	8.0
Osthole	SC	114.19	8	959.19	7.9
	IA	100.48	9	812.61	8.1
Cinnamaldehyde	SC	128.67	7	1103.34	7.7
	IA	125.88	8	1066.11	7.9
Imperatorin	SC	780.37	8	6538.18	8.0
	IA	778.49	9	6452.20	8.1

Notes: Pharmacokinetic parameters were calculated using non-compartmental analysis based on the trapezoidal rule. *AUC*_0–12_ and *MRT*_0–12_ represent partial local exposure within the 12 h microdialysis sampling window. *T_max_* indicates the time to reach the maximum concentration.

## Data Availability

The data supporting the findings of this study are available from the corresponding author upon reasonable request.

## Supplementary Materials

The following supporting information can be downloaded at: https://www.mdpi.com/article/10.3390/pharmaceutics18050524/s1. Table S1: Calibration curves for four analytes in vitro. Table S2: Precision, accuracy, stability, and recovery of the four analytes in vitro. Table S3: Calibration curves for four analytes in microdialysis samples. Table S4: Precision, stability, and recovery of the four analytes in microdialysis samples. Table S5: Batch-level microstructural quantification of GP (mean ± SD, *n* = 6 batches); Table S6: Cumulative release percentages (%) of marker compounds from GP and API groups (mean ± SD, *n* = 6 batches); Table S7: Regression analysis of different kinetic models for in vitro drug release from GP and API groups (*n* = 6 batches); Table S8: K–P model fitting parameters for in vitro drug release of marker compounds from GP and API groups (first 60% cumulative release) (mean ± SD, *n* = 6 batches); Table S9: Cumulative skin permeation percentages (%) of marker compounds from GP (mean ± SD, *n* = 6 batches); Table S10: Regression analysis of different kinetic models for ex vivo skin permeation of GP compounds (*n* = 6 batches); Table S11: ATR–FTIR characteristic peak positions of skin samples in the CON, Vehicle, and GP groups (mean ± SD, *n* = 6 batches); Table S12: Lipid and keratin thermal transition parameters of skin samples in the CON, Vehicle, and GP groups determined by DSC (mean ± SD, *n* = 6 batches); Table S13: Concentration–time data of sinomenine, osthole, cinnamaldehyde, and imperatorin in subcutaneous (SC) tissue and intra-articular (IA) cavity following topical administration of GP (mean ± SD, *n* = 6); Table S14: In vitro–in vivo correlation (IVIVC) results of marker compounds.

## Abbreviations

The following abbreviations are used in this manuscript:

GP	Goupi plaster
RSD	Relative standard deviation
FESEM	Field-emission scanning electron microscopy
ATR–FTIR	Attenuated total reflectance–Fourier transform infrared spectroscopy
DSC	Differential scanning calorimetry
UPLC–MS/MS	Ultra-performance liquid chromatography–tandem mass spectrometry
IVIVC	in vitro–in vivo correlation
SC	Subcutaneous
IA	Intra-articular
API	Active pharmaceutical ingredient
ROI	Region of interest
ESI	Electrospray ionization
MRM	Multiple reaction monitoring
*Q_R_*	Cumulative release amount per unit area
*Q_n_*	Cumulative permeation amount per unit area
*J_ss_*	Steady-state flux
*t_lag_*	Lag time
*P_skin_*	Apparent skin permeability coefficient
AICc	Corrected Akaike information criterion
OLS	Ordinary least squares
CI	Confidence interval
PK	Pharmacokinetics
PD	Pharmacodynamics
