# Supplementary material for: Microstructural Characterization and In Vitro–In Vivo Evaluation of Drug Release and Permeation in Goupi Plaster
Source: Pharmaceutics. 2026 Apr 25;18(5):524. doi: 10.3390/pharmaceutics18050524 (PMC13209900; doi:10.3390/pharmaceutics18050524)
Supplement: Supplementary file 1 [file pharmaceutics-18-00524-s001.zip › pharmaceutics-4221371-supplementary.pdf]

Table S1. Calibration curves for four analytes *in vitro*.

Analyte	Calibration equation	R^2	Linear range (μg/mL)
Sinomenine	y = 7778.70 x − 4511.70	0.9999	0.01–52.00
Osthole	y = 204.07 x + 6.67	0.9992	0.50–100.50
Cinnamaldehyde	y = 611.20 x + 76.28	0.9995	1.00–78.00
Imperatorin	y = 9204.80 x + 5332.00	0.9997	2.00–125.00

Table S2. Precision, accuracy, stability, and recovery of the four analytes *in vitro*.

Analyte	Precision (RSD, %)	Repeatability (RSD, %)	Stability (RSD, %)	Recovery (%)	Recovery RSD (%)
Sinomenine	0.87	0.60	0.55	98.12–100.75	0.85
Osthole	0.99	1.47	1.45	97.92–100.30	0.71
Cinnamaldehyde	0.57	1.06	0.65	98.05–100.10	0.56
Imperatorin	0.18	0.73	0.45	98.20–100.50	0.65

Table S3. Calibration curves for four analytes in microdialysis samples.

Analyte	Calibration equation	R^2	Linear range (ng/mL)
Sinomenine	y = 2.9801x + 23.30	0.9991	1.00–251.00
Osthole	y = 0.4426x + 16.39	0.9994	0.60–425.00
Cinnamaldehyde	y = 0.9433x + 9.66	0.9996	1.00–312.00
Imperatorin	y = 7.9012x + 193.73	0.9989	1.69–810.00

Table S4. Precision, stability, and recovery of the four analytes in microdialysis samples.

Analyte	Precision (RSD, %)	Stability (RSD, %)	Recovery (%)	Recovery RSD (%)
Sinomenine	0.98	1.29	98.02–100.37	1.01
Osthole	0.73	1.06	99.02–100.17	0.49
Cinnamaldehyde	1.09	0.68	98.56–100.21	1.76
Imperatorin	0.32	0.97	99.67–100.05	1.28

Table S5. Batch-level microstructural quantification of GP (mean ± SD, n = 6 batches).

Batch	Porosity	Fiber area fraction	Fiber length density (μm^{-1})
1	0.8168 ± 0.0725	0.0725 ± 0.1779	0.0466 ± 0.0631
2	0.7269 ± 0.0471	0.0471 ± 0.2017	0.0248 ± 0.0737
3	0.7087 ± 0.0453	0.0453 ± 0.2615	0.0255 ± 0.0958
4	0.7451 ± 0.0678	0.0678 ± 0.2214	0.0521 ± 0.0822
5	0.7862 ± 0.0227	0.0227 ± 0.2555	0.0234 ± 0.0934
6	0.6839 ± 0.0907	0.0907 ± 0.1632	0.0321 ± 0.0561

Table S6. Cumulative release percentages (%) of marker compounds from GP and API groups (mean ± SD, n = 6 batches).

Time (h)	Sinomenine (GP)	Osthole (GP)	Cinnamaldehyde (GP)	Imperatorin (GP)	Sinomenine (API)	Osthole (API)	Cinnamaldehyde (API)	Imperatorin (API)
1	9.68 ± 0.46	10.26 ± 0.68	11.06 ± 0.43	10.30 ± 0.55	23.00 ± 3.23	10.20 ± 0.33	21.80 ± 3.22	11.17 ± 3.34
2	15.38 ± 0.82	16.06 ± 0.95	17.34 ± 0.46	21.81 ± 1.11	35.50 ± 4.75	14.15 ± 0.65	34.71 ± 5.24	15.52 ± 4.50
4	23.55 ± 0.98	25.95 ± 0.86	27.80 ± 0.84	33.36 ± 1.50	46.50 ± 5.31	19.60 ± 2.35	41.30 ± 4.89	20.10 ± 4.25
6	30.72 ± 1.17	33.84 ± 1.12	35.37 ± 0.51	35.63 ± 1.44	62.82 ± 5.49	23.70 ± 4.55	56.14 ± 6.40	24.64 ± 5.76
8	35.78 ± 1.16	39.64 ± 1.22	41.25 ± 0.63	40.55 ± 1.01	71.80 ± 3.76	27.10 ± 5.24	67.05 ± 5.65	29.25 ± 5.68
10	44.78 ± 1.56	48.58 ± 1.60	50.71 ± 1.34	53.73 ± 1.74	79.20 ± 3.24	30.10 ± 4.38	76.53 ± 2.95	33.46 ± 4.22
12	47.60 ± 1.72	52.37 ± 1.84	54.82 ± 1.90	59.35 ± 1.92	83.60 ± 4.63	32.90 ± 5.10	80.28 ± 5.72	36.70 ± 5.40
24	76.94 ± 1.68	84.58 ± 0.73	80.10 ± 2.06	81.35 ± 3.25	88.67 ± 4.97	45.50 ± 3.24	83.72 ± 2.83	48.71 ± 5.46

Notes: GP = GP group; API = API group. Data are presented as mean ± SD based on six independent GP batches (n = 6). For each batch, values were obtained by averaging three parallel Franz diffusion cells.

Table S7. Regression analysis of different kinetic models for *in vitro* drug release from GP and API groups (n = 6 batches).

Compound	Model	R^2	AICc	Best-fitting model
Sinomenine (GP)	Zero-order	0.9442	−43.03	Higuchi
	First-order	0.9839	−53.00	
	Higuchi	0.9977	−67.46	
Osthole (GP)	Zero-order	0.9747	−48.42	Higuchi
	First-order	0.9832	−55.34	
	Higuchi	0.9925	−56.87	
Cinnamaldehyde (GP)	Zero-order	0.9754	−50.27	Higuchi
	First-order	0.9797	−51.80	
	Higuchi	0.9908	−56.87	
Imperatorin (GP)	Zero-order	0.9147	−39.52	Higuchi
	First-order	0.9566	−45.08	
	Higuchi	0.9824	−60.78	
Sinomenine (API)	Zero-order	0.7087	−27.37	First-order
	First-order	0.9795	−48.60	
	Higuchi	0.8807	−34.51	
Osthole (API)	Zero-order	0.9438	−52.70	Higuchi
	First-order	0.9288	−50.82	
	Higuchi	0.9998	−97.32	
Cinnamaldehyde (API)	Zero-order	0.7113	−28.09	First-order
	First-order	0.9612	−44.14	
	Higuchi	0.8753	−34.80	
Imperatorin (API)	Zero-order	0.9355	−50.22	Higuchi
	First-order	0.9379	−50.52	
	Higuchi	0.9953	−71.22	

Notes: R^2 = coefficient of determination; AICc = corrected Akaike information criterion. The lowest AICc and highest R^2 indicate the best-fitting model for each compound. GP = GP group; API = API group.

Table S8. K–P model fitting parameters for *in vitro* drug release of marker compounds from GP and API groups (first 60% cumulative release) (mean ± SD, n = 6 batches).

Compound	*k* Value (h^{-n})	*n* Value	Release Mechanism
Sinomenine (GP)	0.1116 ± 0.0029	0.6491 ± 0.1060	Non-Fickian anomalous diffusion
Osthole (GP)	0.1031 ± 0.0033	0.6596 ± 0.0058	Non-Fickian anomalous diffusion
Cinnamaldehyde (GP)	0.0962 ± 0.0024	0.6443 ± 0.0036	Non-Fickian anomalous diffusion
Imperatorin (GP)	0.1269 ± 0.0049	0.6115 ± 0.0541	Non-Fickian anomalous diffusion
Sinomenine (API)	0.2421 ± 0.0378	0.4805 ± 0.0066	Fickian diffusion
Osthole (API)	0.1020 ± 0.0067	0.4705 ± 0.0263	Fickian diffusion
Cinnamaldehyde (API)	0.2270 ± 0.0240	0.4897 ± 0.0200	Fickian diffusion
Imperatorin (API)	0.1095 ± 0.0258	0.4737 ± 0.0185	Fickian diffusion

Notes: *k* = rate constant of the Korsmeyer–Peppas model; *n* = release exponent. GP = GP group; API = API control group.

Table S9. Cumulative skin permeation percentages (%) of marker compounds from GP (mean ± SD, n = 6 batches)

Time (h)	Sinomenine	Osthole	Cinnamaldehyde	Imperatorin
1	5.64 ± 1.12	4.77 ± 0.82	4.94 ± 0.31	5.60 ± 0.37
2	11.09 ± 1.16	10.81 ± 0.64	9.19 ± 0.53	10.78 ± 0.73
4	18.98 ± 1.94	16.31 ± 1.74	16.04 ± 0.60	14.31 ± 1.32
6	19.97 ± 1.99	20.45 ± 2.50	20.41 ± 0.75	17.37 ± 0.97
8	26.17 ± 1.23	24.37 ± 1.98	23.77 ± 0.68	21.98 ± 0.68
10	30.88 ± 2.39	27.94 ± 2.42	26.10 ± 0.98	28.21 ± 1.17
12	35.80 ± 1.81	31.77 ± 1.24	27.63 ± 1.09	31.98 ± 1.29
24	58.63 ± 1.51	56.96 ± 3.99	51.58 ± 0.93	54.62 ± 2.18

Table S10. Regression analysis of different kinetic models for *ex vivo* skin permeation of GP compounds (n = 6 batches).

Compound	Model	R^2	AICc	Best-fitting model
Sinomenine	Zero-order	0.9821	−56.47	Zero-order
	First-order	0.9802	−53.52	
	Higuchi	0.9813	−54.88	
Osthole	Zero-order	0.9895	−60.46	Zero-order
	First-order	0.9783	−54.19	
	Higuchi	0.9738	−53.20	
Cinnamaldehyde	Zero-order	0.9728	−57.89	Zero-order
	First-order	0.9657	−55.34	
	Higuchi	0.9734	−55.67	
Imperatorin	Zero-order	0.9927	−63.97	Zero-order
	First-order	0.9818	−58.72	
	Higuchi	0.9665	−51.72	

Notes: R^2 = coefficient of determination; AICc = corrected Akaike information criterion. The lowest AICc and highest R^2 indicate the best-fitting model for each compound.

Table S11. ATR–FTIR characteristic peak positions of skin samples in the CON, Vehicle, and GP groups (mean ± SD, n = 6 batches).

Group	$\nu asCH_2$ (cm^{-1})	νsCH_2 (cm^{-1})	Amide I (cm^{-1})	Amide II (cm^{-1})
CON group	2920.90 ± 0.55	2850.88 ± 0.62	1631.70 ± 0.32	1550.64 ± 0.47
Vehicle group	2921.28 ± 0.83	2851.37 ± 0.77	1632.56 ± 0.35	1549.03 ± 0.69
GP group	2924.20 ± 0.91	2854.65 ± 0.86	1635.66 ± 0.84	1546.74 ± 0.77

Notes: $\nu asCH_2$ and νsCH_2 represent the asymmetric and symmetric stretching vibrations of methylene groups, respectively.

Table S12. Lipid and keratin thermal transition parameters of skin samples in the CON, Vehicle, and GP groups determined by DSC (mean ± SD, n = 6 batches).

Group	Lipid characteristic peak (°C)	Lipid peak intensity ($mW\cdot mg^{-1}$)	Keratin characteristic peak (°C)	Keratin peak intensity ($mW\cdot mg^{-1}$)
CON group	65.37 ± 0.43	−60.28 ± 0.75	109.69 ± 0.53	−108.48 ± 0.21
Vehicle group	66.99 ± 0.69	−51.18 ± 0.67	110.15 ± 0.78	−92.20 ± 0.42
GP group	69.82 ± 0.63	−29.57 ± 0.25	113.06 ± 0.74	−52.56 ± 0.64

Notes: Lipid and keratin transitions were determined from DSC thermograms of skin samples.

Table S13. Concentration–time data of sinomenine, osthole, cinnamaldehyde, and imperatorin in subcutaneous (SC) tissue and intra-articular (IA) cavity following topical administration of GP (mean ± SD, n = 6).

Time (h)	Sinomenine (SC, ng·mL^{-1})	Sinomenine (IA, ng·mL^{-1})	Osthole (SC, ng·mL^{-1})	Osthole (IA, ng·mL^{-1})	Cinnamaldehyde (SC, ng·mL^{-1})	Cinnamaldehyde (IA, ng·mL^{-1})	Imperatorin (SC, ng·mL^{-1})	Imperatorin (IA, ng·mL^{-1})
1	7.42 ± 3.32	3.68 ± 1.68	0.83 ± 0.49	——	4.48 ± 0.80	——	14.42 ± 3.16	7.94 ± 2.09
2	51.88 ± 4.15	44.79 ± 2.33	49.46 ± 2.21	21.60 ± 4.10	15.91 ± 1.37	2.55 ± 0.69	187.51 ± 6.95	153.92 ± 5.37
3	92.48 ± 2.90	68.27 ± 3.81	77.34 ± 2.04	45.28 ± 3.70	27.12 ± 1.53	9.30 ± 1.06	326.37 ± 8.73	292.94 ± 6.08
4	130.25 ± 2.39	101.23 ± 3.32	115.74 ± 4.85	67.25 ± 6.00	41.08 ± 4.19	20.56 ± 2.17	461.95 ± 8.09	432.91 ± 4.49
5	169.87 ± 2.59	134.46 ± 3.17	163.73 ± 2.08	103.49 ± 3.93	56.24 ± 2.84	32.43 ± 3.85	573.18 ± 5.57	545.26 ± 5.92
6	209.58 ± 3.82	158.62 ± 2.48	201.47 ± 2.21	139.24 ± 4.00	72.75 ± 4.34	43.48 ± 2.34	661.67 ± 6.42	632.91 ± 8.38
7	247.63 ± 2.60	195.92 ± 3.61	238.48 ± 9.18	175.60 ± 7.70	85.16 ± 2.68	54.52 ± 3.84	743.84 ± 8.79	715.36 ± 8.33
8	254.34 ± 6.69	210.82 ± 5.47	246.65 ± 2.19	195.55 ± 4.10	95.02 ± 2.35	62.41 ± 2.67	780.37 ± 6.39	745.16 ± 5.35
9	250.18 ± 4.02	226.34 ± 3.47	242.66 ± 3.59	211.38 ± 9.02	94.75 ± 4.52	66.41 ± 5.32	779.16 ± 9.03	768.49 ± 6.28
10	245.15 ± 2.62	218.69 ± 3.66	235.64 ± 4.17	203.42 ± 4.00	90.26 ± 2.83	62.42 ± 2.23	777.94 ± 5.11	760.32 ± 8.11
11	234.73 ± 2.43	211.92 ± 4.73	225.71 ± 4.84	197.87 ± 4.98	84.61 ± 6.66	58.91 ± 4.07	767.61 ± 4.24	755.62 ± 6.05
12	224.47 ± 5.31	204.14 ± 2.83	215.25 ± 5.89	194.38 ± 4.00	78.10 ± 3.85	55.41 ± 2.84	762.74 ± 5.69	753.67 ± 6.86

Notes: Data are presented as mean ± SD (n = 6). SC = subcutaneous tissue; IA = intra-articular cavity.

Table S14. ***In vitro–in vivo*** **correlation (IVIVC) results of marker compounds.**

Compound		Sinomenine	Osthole	Cinnamaldehyde	Imperatorin
In vitro release vs SC	Slope	6.4070 ± 0.6770	17.4555 ± 0.6356	2.3259 ± 0.0566	7.5485 ± 0.8011
	R^2	0.8957 ± 0.0366	0.8991 ± 0.0640	0.9273 ± 0.0402	0.9633 ± 0.0547
In vitro release vs IA	Slope	5.8543 ± 0.6000	18.4179 ± 0.8689	1.8011 ± 0.0963	5.1534 ± 0.3580
	R^2	0.9493 ± 0.0287	0.9253 ± 0.0967	0.9326 ± 0.0216	0.8674 ± 0.0413
Ex vivo permeation vs SC	Slope	11.2049 ± 0.4001	32.4827 ± 0.3035	3.4712 ± 0.0758	11.4997 ± 0.9580
	R^2	0.9641 ± 0.0333	0.8732 ± 0.0393	0.9086 ± 0.0465	0.9690 ± 0.0391
Ex vivo permeation vs IA	Slope	9.5299 ± 0.3384	35.4063 ± 0.3060	2.8612 ± 0.0980	9.1243 ± 0.7883
	R^2	0.9857 ± 0.01220	0.9038 ± 0.0506	0.8906 ± 0.0209	0.8524 ± 0.0309

Note: R^2 = coefficient of determination.
